# Resveratrol ameliorates renal injury in spontaneously hypertensive rats by inhibiting renal micro-inflammation

**DOI:** 10.1042/BSR20160035

**Published:** 2016-06-03

**Authors:** Hai-Yan Xue, Li Yuan, Ying-Jie Cao, Ya-Ping Fan, Xiao-Lan Chen, Xin-Zhong Huang

**Affiliations:** * Department of Nephrology, Affiliated Hospital of Nantong University, Nantong, Jiangsu 226001, China

**Keywords:** inflammation, intercellular adhesion molecule-1 (ICAM-1), interleukin-6 (IL-6), monocyte chemoattractant protein 1 (MCP-1), nuclear factor κB (NF-κB), resveratrol (RSV), spontaneously hypertensive rat (SHR)

## Abstract

Micro-inflammation plays an important role in the pathogenesis of spontaneously hypertensive rat (SHR). In the present study, we investigated the therapeutic potential of resveratrol (RSV), a polyphenol with anti-fibrosis activity in hypertensive renal damage model. In SHR renal damage model, RSV treatment blunted the increase in urine albumin excretion, urinary β_2_-microglobulin (β_2_-MG), attenuated the decrease in creatinine clearance rate (CCR). The glomerular sclerosis index (1.54±0.33 compared with 0.36±0.07) and tubulointerstitial fibrosis (1.57±0.31 compared with 0.19±0.04) were significantly higher in SHRs compared with Wistar Kyoto rats (WKYs), which were significantly lower by RSV treatment. The increases in mesangium accumulation and the expression of renal collagen type I (Col I), fibronectin (Fn), plasminogen activator inhibitor-1 (PAI-1) and transforming growth factor-β1 (TGF-β1) in SHR were also reduced by RSV treatment. Nuclear factor κB (NF-κB) expression was increased in the cytoplasm and nuclei of the SHR kidneys, which was significantly decreased by RSV treatment. Furthermore, the protein level of IκB-α significantly decreased in the kidneys of the SHR when compared with the WKYs. RSV treatment partially restored the decreased IκB-α level. In SHR kidney, increased expression of interleukin-6 (IL-6), intercellular adhesion molecule-1 (ICAM-1) and monocyte chemoattractant protein 1 (MCP-1) were observed. These changes were attenuated by RSV treatment. No changes in blood pressure were detected between SHR group and SHR + RSV group. Taken together, the present study demonstrated that RSV treatment may significantly attenuate renal damage in the SHR model of chronic kidney disease (CKD). The renal protective effect is associated with inhibition of IL-6, ICAM-1 and MCP-1 expression via the regulation of the nuclear translocation of NF-κB, which suggesting that micro-inflammation may be a potential therapeutic target of hypertensive renal damage.

## INTRODUCTION

It is well-documented that chronic kidney disease (CKD) is a life-threatening disease frequently associated with hypertension, progression to renal fibrosis and eventual renal failure [1–4]. It affects 26 million American adults and is the ninth leading cause of mortality in the United States [5,6]. In the past decades, although numerous studies have been performed aiming to develop better strategies for treating the CKDs, the therapeutic outcome is still unsatisfactory owing to the incomplete understanding of pathological mechanisms [7,8]. This situation raised an urgent request to better understand the pathogenic mechanisms of CKDs.

Recently, an increased inflammatory response is known to be associated with the disease and has long been speculated to contribute to disease development [9,10]. For example several recent studies have shown that micro-inflammatory cytokines in patients with hypertension were significantly higher than normal, and further increase with the degree of renal dysfunction aggravate, which indicates that micro-inflammatory cytokines involved in the pathological process of developing hypertension renal damage [11–15]. Nuclear factor κB (NF-κB), an inflammatory transcription factor, plays an important role in the pathogenesis of hypertensive nephropathy. Inactive NF-κB is sequestered within the cytoplasm and bound by the members of the IκB family of inhibitor proteins. The various stimuli that activate NF-κB cause the phosphorylation of IκB, which is followed by its ubiquitination and subsequent degradation. Finally, NF-κB migrates to the nucleus and activates the transcription of several target genes, including interleukin-6 (IL-6), intercellular adhesion molecule-1 (ICAM-1) and monocyte chemoattractant protein 1 (MCP-1). These genes induce persistent kidney micro-inflammation [16,17]. Spontaneously hypertensive rat (SHR), the most widely used animal model of human essential hypertension, gradually develop progressive glomerular sclerosis and interstitial fibrosis, preceded by a renal inflammatory infiltrate that is thought to play an important role in the genesis of renal injury [18–21].

Resveratrol (RSV; 3,5,4′-trihydroxystilbene) is one of the natural polyphenols compound with anti-inflammatory, antioxidant, anti-atherosclerotic, anti-fibrosis properties, which can be available in red wine, pomegranates, and Polygonum Cuspidatum used in traditional Chinese medicine [22–24]. RSV has various health beneficial effects on cardiovascular diseases, cerebral ischaemic injuries, aging and cancer [25]. RSV is reported to suppress angiotensin II (Ang II)-induced Akt activation and extracellular signal-regulated protein kinase to a lesser extent [26]. Our previous and recent studies have demonstrated that RSV treatment significantly ameliorates proteinuria and renal fibrosis in subtotal nephrectomized rats by inhibiting Smad3 acetylation and attenuates urine albumin excretion, glomerular basement membrane thickness and renal fibrosis in diabetic nephropathy via modulating angiogenesis [22,27]. However, the effect of RSV on hypertensive renal damage remains to be determined.

The aim of the present study is to examine whether RSV ameliorates micro-inflammatory cytokines including IL-6, ICAM-1 and MCP-1 expression and protect hypertensive renal damage by inhibiting NF-κB pathway. The present study provides important experimental evidence supporting RSV as a potential therapeutic drug for hypertensive renal damage.

## MATERIALS AND METHODS

### Animal models

All animal studies were approved by the Institutional Animal Care and Use Committees of Nantong University. Male SHRs and normotensive Wistar Kyoto rats (WKYs) were maintained in the animal facility of Nantong University Medical Animal Center, where they were housed in a constant-temperature room with a 12 h dark/12 h light circle and allowed free access to standard rodent chow and water. A total of 20 SHRs at the age of 7 weeks were uninephrectomized from the right side [19]. Welfare-related assessment and interventions were carried out during the experiment. One week later, these SHRs were administered either RSV (20 mg/kg, Copalyton Chemical Materials) (RSV, *n*=10) or vehicle (SHR, *n*=10) via daily oral gavage and continuing for a period of 40 weeks. Vehicle-treated groups (WKY, *n*=10), which uninephrectomized from the right side, received an equal volume of normal saline. At 10-week intervals, arterial blood pressure was measured in conscious, restrained rats using a tail-cuff system (RBP-1 system) in a dark temperature-controlled room (22°C) in the morning. Rats were subjected to 15 acclimation measurements in restraint holder then blood pressure was calculated from the average of ten measurement cycles. At the age of 48 weeks, their urine samples were collected in individual metabolic cages, and then their blood was collected by cardiac puncture after anesthetized and their kidney tissue samples were collected for immunohistochemistry, Western blot and real-time PCR as described recently [22].

### Urine and serum biochemical tests

Urine albumin and β_2_-microglobulin (β_2_-MG) were determined by biuret method and radio-immunity kits respectively. Rats’ urine and serum creatinine levels were measured using an automated analyser according to the manufacturer's instructions.

### Histological examination

Tissues fixed in 4% paraformaldehyde/PBS were embedded in paraffin using routine protocols. Paraffin-embedded materials were sectioned at 3 μm for routine staining with haematoxylin/eosin, periodic acid-Schiff (PAS) and Masson trichrome. Glomerulosclerosis was assessed in 50 glomeruli on PAS-stained sections under ×400 magnification using a semi-quantitative score from 0 to 4 (0, no sclerosis; 1, sclerosis up to 25% of glomeruli; 2, sclerosis from 25% to 50% of glomeruli; 3, sclerosis from 50% to 75% of glomeruli; 4, sclerosis >75% of glomeruli), and the results were averaged. For evaluating tubulointerstitial damage, 15 fields for each section (Masson trichrome stain) were evaluated at ×200 magnification using Image-Pro Plus 6.0 processing software (Media Cybernetics). The extent of tubulointerstitial damage was evaluated by counting the percentage of areas with tubular dilation, interstitial infiltration and fibrosis per field of cortex. Scores from 0 to 4 were used (0, normal interstitium; 1, <25% of areas injured; 2, 26%–50% of areas injured; 3, 51%–75% of areas injured; 4, >75% of areas injured), and the results were averaged. The number of inflammatory cells that infiltrated the renal interstitial area was determined by counting nuclei in haematoxylin/eosin-stained sections. All histological analyses were performed by two investigators without knowledge of the origin of the slides, and the mean values were calculated.

### Immunohistochemistry

For immunohistochemistry examination, kidney sections were immunostained using immunoperoxidase technique with Vector ABC kit (Vector Laboratories). Briefly, sections (3μm thick) were blocked with 3% BSA for 30 min at room temperature, and incubated with primary antibody overnight at 4°C. The primary antibodies used were anti-NF-κB p65 antibody (Santa Cruz Biotechnology mouse monoclonal, 1:200), anti-IL-6 antibody (Santa Cruz Biotechnology rabbit polyclonal, 1:200) and anti-ICAM-1 antibody (Santa Cruz Biotechnology mouse monoclonal, 1:200). Sections were then washed and incubated with biotinylated secondary antibodies for 60 min at room temperature. Biotin was identified and visualized with 3,3’-diaminobenzidine (DAB) solution. As a negative control, the primary antibody was replaced with nonimmune IgG, and no staining occurred. Counterstaining was then performed before examination under a light microscope. Random 100 glomeruli from each renal specimen were observed, and images were then analysed with Image Pro Plus 6.0 edition (Media Cybernetics) for the determination of immunostained area. The percentage of the stained area was calculated as the ratio of suitable binary thresholded image and the total field area.

### Real-time reverse transcription-PCR

Total RNA was extracted from tissues using TRizol reagent (Invitrogen). RNA was subjected to reverse transcription (RT) with reverse transcriptase as per manufacturer's instructions (Fermentas). Quantitative real-time PCR was performed using the Bio-Rad iQ5 system and the relative gene expression was normalized to internal control using β-actin. Primer sequences for SYBR Green probes of target genes are shown as Table 1. The amplification efficiency and specificity were confirmed before applying them to the assay.

**Table 1:** Primer sequences for SYBR Green probes of target genes

Name	Primer sequence (5’→3’)
Col I-F	ATCCTGCCTATGTCGCTAT
Col I-R	CCACAAGCGTGCTGTAGGT
FN-F	TCGCTTTGACTTCACCACCAG
FN-R	CCTCGCTCAGTTCGTACTCCAC
IL-6-F	TTGCCTTCTTGGGACTGATG
IL-6-R	ATGACTCTGGCTTTGTCTTTCT
ICAM-1-F	CCTGGGTCATAATTGTTGGTG
ICAM-1-R	AGGAAGTCAGCCTTTCTTGG
MCP-1-F	GCTGCTACTCATTCACTGGCAA
MCP-1-R	TGCTGCTGGTGATTCTCTTGTA
β-Actin-F	CCCATACCCACCATCACACC
β-Actin-R	GAGAGGGAAATCGTGCGTGAC

### Western blot analysis

Protein from kidney tissues was extracted with RIPA lysis buffer, and Western blot analysis was performed as described recently [22]. Protein concentration was determined using the BCA protein assay (Sigma–Aldrich). Fifty micrograms of proteins were loaded in each lane of a SDS/PAGE (10% gel) and run at 100 V. Protein were transferred to nitrocellulose membranes, which were then washed and incubated with blocking buffer (5% non-fat milk in PBS containing 0.1% Tween 20) for 1 h at room temperature. The membranes were then incubated overnight at 4°C with the primary antibodies: collagen type I (Col I) (Abcam rabbit polyclonal, 1:1000), fibronectin (Fn) (Sigma–Aldrich chicken polyclonal, 1:1000), plasminogen activator inhibitor-1 (PAI-1), IL-6 (Santa Cruz Biotechnology rabbit polyclonal, 1:1000), ED-1, ICAM-1 (Santa Cruz Biotechnology mouse monoclonal, 1:1000), MCP-1 (Santa Cruz Biotechnology goat polyclonal, 1:1000), anti-IκB-α antibody (Abcam rabbit monoclonal, 1:200) and β-actin (Sigma–Aldrich mouse monoclonal, 1:2000). After three washes, the membranes were incubated with horseradish-peroxidase-conjugated secondary antibodies (Santa Cruz Biotechnology) for 1 h at room temperature followed by three washes with TBST. Antibody labelling was visualized by the addition of chemiluminescence reagent (Amersham Biosciences), and the membrane was exposed to Kodak XAR-5 film.

### Homogenization of kidney tissues

Kidney tissues (cortex) were homogenized with T-PER mammalian protein extraction regent (20 ml/g renal tissues; Pierce Biotechnology) containing 1% (v/v) protease inhibitor cocktail (Sigma–Aldrich) using a TissueLyser (Qiagen). Harvested lysates were then centrifuged 12000 ***g*** for 10 min at 4°C to remove the cellular debris. The supernatants were collected and stored at −80°C. Protein concentration was measured using the BCA protein assay reagent kit (Pierce Biotechnology).

Measurement of transforming growth factor-β1 (TGF-β1) protein levels were measured in kidney tissue homogenates from each sample using the TGF-β1 ELISA kit (R&D Systems) following the manufacturer′s instructions. To control for the difference between samples, the concentration was corrected based on the amount of total tissue protein.

### Statistical analysis

Statistical software SPSS ver. 15.0 (SPSS) was used to perform data statistical analysis. Data were shown as mean ± S.D. Statistical significance was determined by one-way ANOVA. Differences with *P*<0.05 were considered statistically significant.

## RESULTS

### Effects of RSV on creatinine clearance rate, urinary albumin/creatinine, β_2_-MG/creatinine and arteria caudilis pressure in SHR

**Figure 1:**
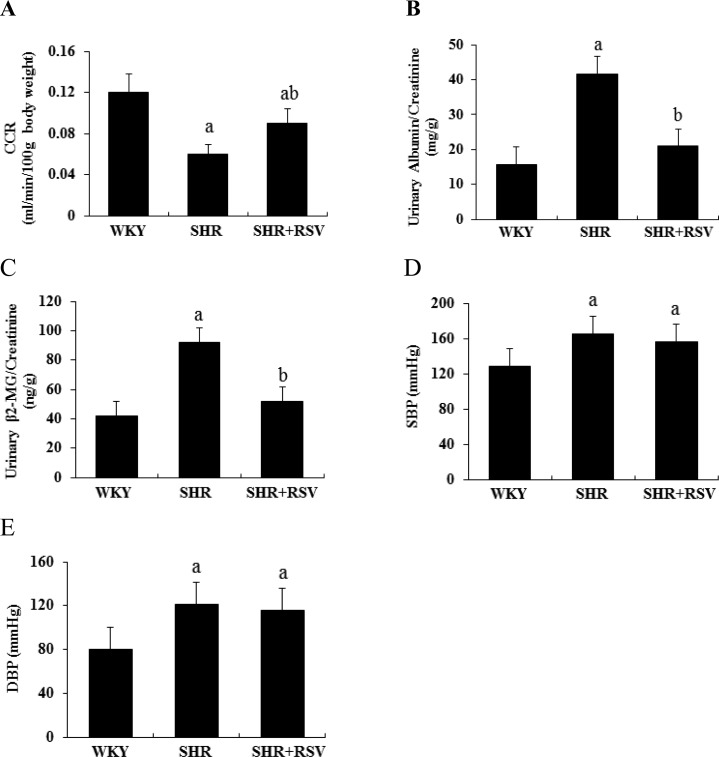
RSV preserves renal function in SHR Effects of RSV on CCR, urinary albumin/creatinine, β_2_-MG/creatinine and arteria caudilis pressure levels at the age of 48 weeks in SHR. (**A**) CCR; (**B**) urinary albumin/creatinine; (**C**) urinary β_2_-MG/creatinine; (**D**) systolic blood pressure; (**E**) diastolic blood pressure. Data are means ± S.D., (a*P*<0.05 compared with WKY; ^b^*P*<0.05 compared with SHR).

Figure 1 presents the renal function including creatinine clearance rate (CCR), urinary albumin/creatinine, β_2_-MG/creatinine and arteria caudilis pressure of SHR and WKY rats treated with or without RSV. SHR significantly decreased CCR and increased the ratio of urinary albumin/creatinine and β_2_-MG/creatinine compared with WKY group. RSV treatment significantly blunted the decrease in CCR and the increase in the ratio of urinary albumin/creatinine and β_2_-MG/creatinine in the SHR rats (Figures 1A and 1C). Arteria caudilis pressure was significantly increased in SHR at the 48th week compared with WKY group. RSV treatment did not reduce the increase in blood pressure in SHR (Figures 1D and 1E).

### RSV attenuates kidney damage in SHR

**Figure 2:**
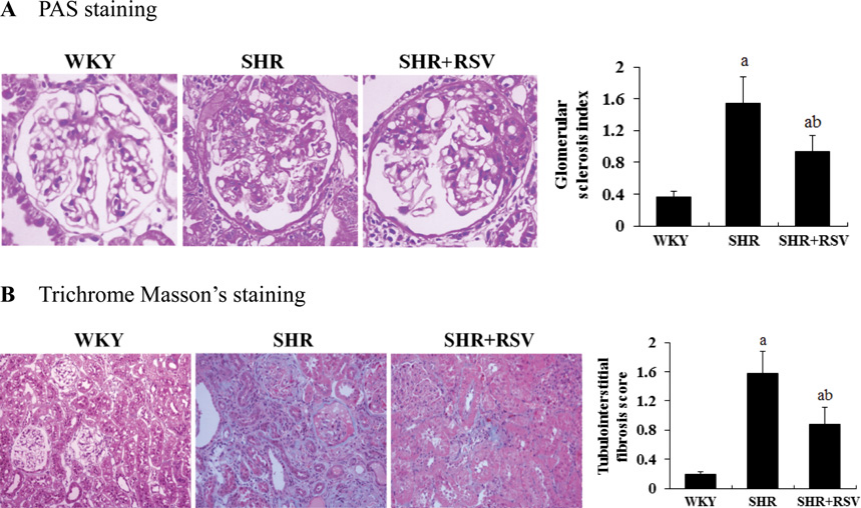
RSV attenuates renal histological changes in SHR Effects of RSV on renal histology in SHR. Light microscopic findings and quantitative analysis in the study groups. Representative pictures stained with (**A**) PAS and (**B**) Masson trichrome in WKY, SHR and SHR + RSV group; a*P*<0.01 compared with WKY; ^b^*P*<0.01 compared with SHR. Magnifications: ×400 in (A); ×200 in (B).

Figures 2(A) and 2(B) represent the PAS and Masson trichrome staining of the kidneys. The kidneys of the SHR were characterized by glomerular sclerosis and tubulointerstitial fibrosis. Semi-quantitative analysis shows that the glomerular sclerosis index (1.54±0.33 compared with 0.36±0.07) and tubulointerstitial fibrosis (1.57±0.31 compared with 0.19±0.04) were significantly increased in SHRs compared with WKYs (*P*<0.01). RSV substantially reversed these changes.

### Effects of RSV on profibrogenic genes and proteins in SHR

**Figure 3:**
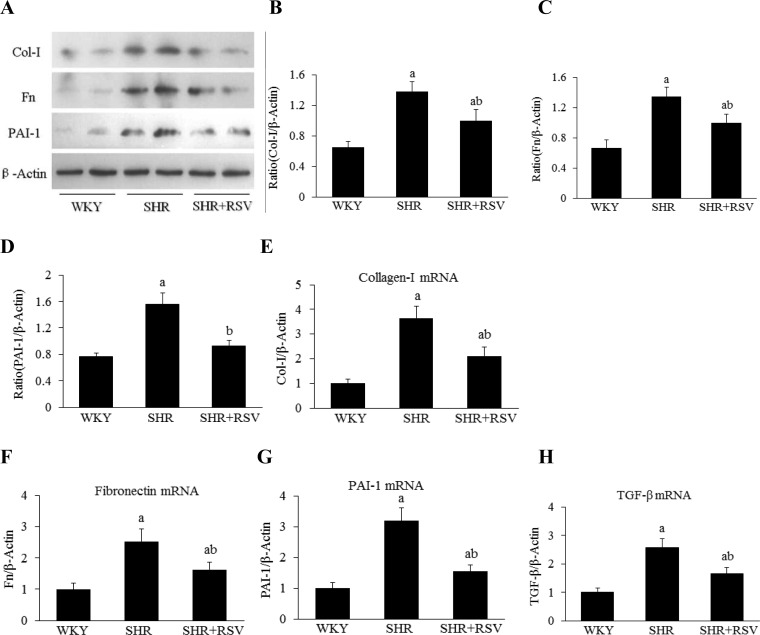
RSV decreases profibrogenic gene expression in SHR Effects of RSV on renal fibrotic gene and protein expression levels in SHR (*n*=6). (**A**) Col I, Fn and PAI-1 expression by Western blot. (**B**–**D**) Quantification of Col I, Fn and PAI-1 expression is achieved using densitometric values normalized to β-actin levels. (**E**–**H**) The relative mRNA levels of Col I, Fn, PAI-1 and TGF-β expression by real-time semi-quantitative PCR. The values were normalized to the β-actin values and then expressed as relative quantification, (a*P*<0.05 compared with WKY; ^b^*P*<0.05 compared with SHR).

**Figure 4:**
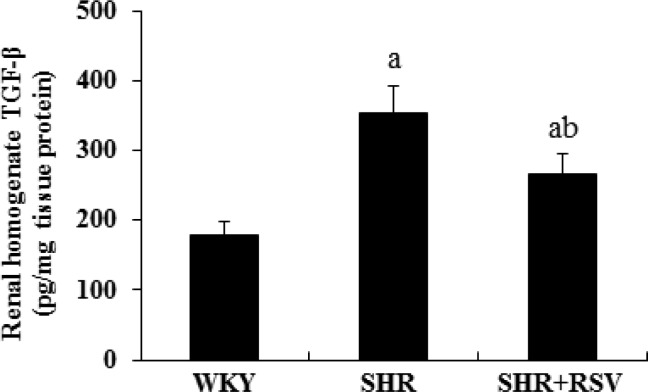
RSV decrease renal cortical TGF-β1 protein level in SHR Renal cortical homogenate TGF-β1 protein levels of each group measured by ELISA. Data are expressed as means ± S.D., (a*P*<0.05 compared with WKY; ^b^*P*<0.05 compared with SHR).

The gene and protein expression level of collagen I was much higher in SHR than in WKY rats, as assessed by real-time RT-PCR and Western blot. RSV treatment significantly decreased *Col I* gene and protein expression in SHRs (Figures 3A, 3B and 3E). There was an increase in Fn and *PAI-1* gene and protein expression in SHRs compared with WKYs. Fn and *PAI-1* gene and protein expression in the kidneys of SHRs were attenuated by RSV treatment (Figures 3A,3C,3D,3F and 3G). As shown in Figure 3(H), there was a significant increase in transforming growth factor-β (TGF-β) gene expressions in the kidneys of SHRs compared with WKYs that was significantly reversed by RSV treatment. For examination of the effect of RSV on TGF-β protein synthesis in SHRs, kidney tissue homogenate was measured using the TGF-β ELISA kit. The TGF-β level was significantly higher in SHRs than in WKYs (*P*<0.05). There was a 25% reduction in TGF-β by RSV treatment in SHRs (Figure 4).

### RSV ameliorates renal inflammation in SHR

**Figure 5:**
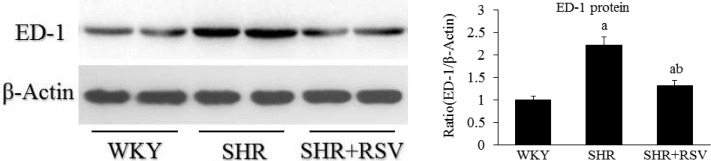
RSV decreases renal ED-1 expression in SHR Renal homogenate ED-1 protein levels of each group measured by Western blot. Data are means ± S.D. The values were normalized to the β-actin values and then expressed as relative quantification, (a*P*<0.05 compared with WKY; ^b^*P*<0.05 compared with SHR).

SHRs demonstrated severe inflammation in both glomeruli and interstitium. This was significantly attenuated by RSV treatment. For confirmation of these findings and avoidance of possible bias in tissue sectioning, selection and counting, immunoblot analysis of ED-1 protein was performed on whole-kidney homogenates, which also showed that kidney ED-1 levels were reduced by RSV (Figure 5).

### Effects of RSV on NF-κB expression in SHR

**Figure 6:**
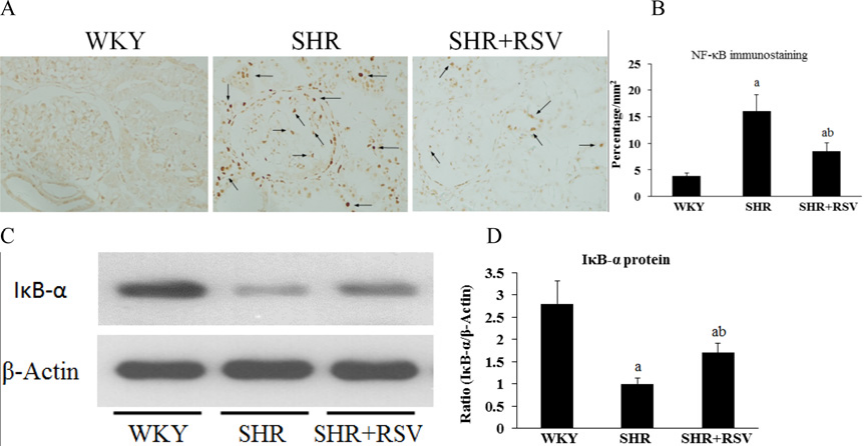
RSV decreases renal NF-κB activity in SHR Effects of RSV on renal NF-κB localization in SHR rats. (**A**–**B**) NF-κB p65 location in each group was detected by immunohistochemistry. Nuclear localization of NF-κB is highlighted by arrows. (**C**–**D**) The protein level of IκB-α in kidney was detected by Western blot analysis. Data are means ± S.D. The values were normalized to the β-actin values and then expressed as relative quantification, (a*P*<0.05 compared with WKY; ^b^*P*<0.05 compared with SHR).

Immunohistology staining for NF-κB p65 demonstrated that a small quantity of NF-κB p65 localized in the cytoplasm of the WKY kidneys and that the nuclei were relatively unstained. By contrast, NF-κB p65 expression was increased in the cytoplasm and nuclei of the SHR kidneys. RSV treatment significantly decreased NF-κB activity in SHR kidneys (*P*<0.05, Figures 6A and 6B). Furthermore, the protein level of IκB-α significantly decreased in the kidneys of the SHR when compared with the WKYs. However, RSV treatment partially restored the decreased IκB-α level (*P*<0.05, Figures 6C and 6D), confirming that the NF-κB pathway is activated in SHR kidneys and that RSV treatment may potentially suppress NF-κB pathway activation in the setting of hypertensive renal injury.

### Effects of RSV on micro-inflammatory genes in SHR

**Figure 7:**
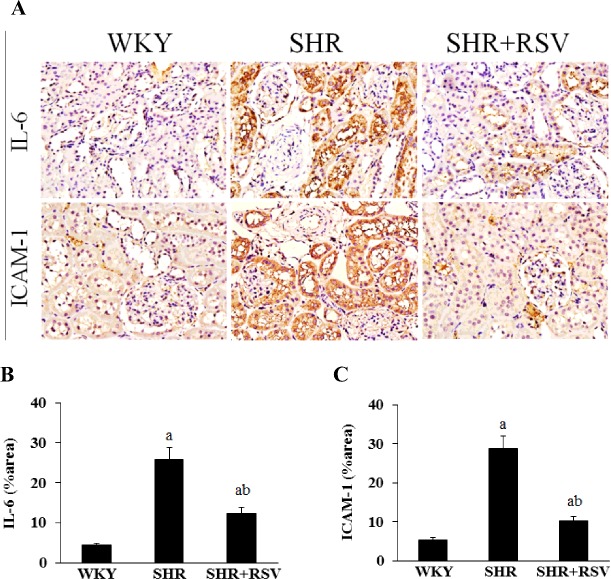
RSV decreases renal IL-6 and ICAM-1 expression in SHR Effects of RSV on renal micro-inflammatory genes expression levels in SHR rats. (**A**) IL-6 and ICAM-1 expression by immunohistochemistry. (**B**–**C**) Quantitative analysis of immunohistochemical staining. Data are means ± S.E.M., (a*P*<0.05 compared with WKY; ^b^*P*<0.05 compared with SHR).

**Figure 8:**
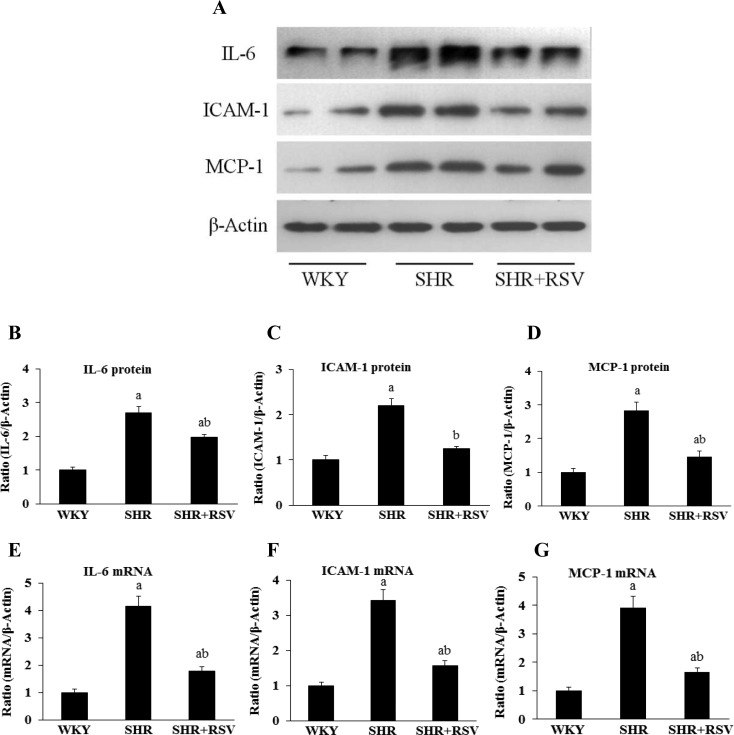
RSV decreases renal IL-6, ICAM-1 and MCP-1 expression in SHR Effects of RSV on renal micro-inflammatory genes expression levels in SHR rats. (**A**) IL-6, ICAM-1 and MCP-1 expression by Western blot. (**B**–**D**) Quantification of IL-6, ICAM-1 and MCP-1 expression is achieved using densitometric values normalized to β-actin levels. (**E**–**G**) IL-6, ICAM-1 and MCP-1 expression by real-time semi-quantitative PCR. Data are means ± S.E.M. The values were normalized to the β-actin values and then expressed as relative quantification (a*P*<0.05 compared with WKY; ^b^*P*<0.05 compared with SHR).

Because macrophage-derived micro-inflammatory cytokines are fundamental in the pathogenesis of hypertensive renal damage, they were examined by real-time RT-PCR, Western blot and immunohistochemistry. The gene and protein expression levels of IL-6, ICAM-1 and MCP-1, which are known as macrophage-associated micro-inflammatory cytokines, were much higher in SHRs than in WKYs. Consistent with the reduction in macrophage infiltration, RSV treatment significantly decreased gene and protein expression levels of IL-6, ICAM-1 and MCP-1 in SHR rats (Figures 7 and 8).

## DISCUSSION

In the present study, we found that RSV decreased urinary albumin and β_2_-MG excretion, attenuated renal pathological changes, reduced expression of renal Fn, Col I and PAI-1, inhibited NF-κB activity and expression of renal IL-6, ICAM-1 and MCP-1, each of which have been found, as here, to be elevated in the SHR. These findings indicate that RSV may be a potential therapeutic drug in hypertensive renal damage via the regulation of the nuclear translocation of NF-κB and micro-inflammatory mediators.

The SHR is the most widely used animal model of human essential hypertension. Hypertensive kidney damage is not morphologically evident before 30 weeks of age in SHRs [28–30]. The damage then progresses slowly with increasing age, in a process similar to human hypertensive renal damage. In the present study, we selected 48-week-old uninephrectomized SHRs as subjects, which have seldom been studied. Compared with the age matched WKYs, increased urinary albumin and β_2_-MG excretion and more extensive profibrogenic genes and proteins expression were observed, gradually develop progressive glomerular sclerosis and interstitial fibrosis.

RSV, a polyphenolic compound found in red wine, has various health beneficial effects through JNK/NF-κB, p38, NO/NOS pathway [31,32]. Li et al. [33] found that RSV can improve renal interstitial fibrosis by specifically inhibiting Smad3 acetylation in unilateral ureteral obstruction mice. Several pieces of evidence from our recent study suggest that RSV could decrease proteinuria and serum creatinine, attenuate renal pathological change and the expression of renal fibrosis gene through Sirt1 activation and reduced Smad3 acetylation [22]. That study also provides strong evidence that RSV can decrease urinary albumin and β_2_-MG excretion, attenuates renal pathological changes, reduce the expression of renal Fn, Col I and PAI-1. These results suggest that RSV is an anti-fibrotic factor and a potential therapeutic drug for hypertension renal damage. RSV treatment did not significantly alter the blood pressure. These data suggest that the beneficial effect of RSV is not haemodynamically mediated. We therefore examined other factors that might account for this effect.

Inflammation plays a detrimental role in the occurrence and development of kidney injury in various kidney diseases including CKDs. Both infiltrating inflammatory cells and renal resident cells contribute to inflammatory response in CKD [34–36]. Numerous studies have demonstrated that tumor necrosis factor-α (TNF-α), MCP-1 and interleukin-1β (IL-1β) participate in different inflammatory states associated with renal diseases, including ischaemia/reperfusion injury [37], kidney transplantation [38], diabetic nephropathy [39] and hypertensive renal damage [40]. However, the detailed mechanisms leading to the inflammation in this model are still poorly understood.

It is well-documented that IL-6, ICAM-1 and MCP-1 are multifunctional micro-inflammatory cytokines involved in immune and inflammatory responses produced by a variety of cells *in vivo* [41,42]. Recent and previous studies demonstrated that the plasma IL-6, ICAM-1, C-reactive protein (CRP) and TNF-α level increased in patients with hypertension, and further rise with the degree of renal damage, suggesting that micro-inflammatory cytokines involved in the development of hypertensive renal damage [43,44]. The present study demonstrates that macrophage accumulation and the expression of mRNA and protein of IL-6, ICAM-1 and MCP-1 were significantly elevated in SHR group. RSV treatment significantly attenuates the damage of renal pathologic changes and inhibition of renal fibrosis, which is associated with down-regulation of macrophage accumulation and the expression of IL-6, ICAM-1 and MCP-1. These results suggest that RSV is an anti-fibrotic factor and a potential therapeutic drug for hypertensive renal damage.

As micro-inflammatory mediators IL-6, ICAM-1 and MCP-1 are downstream products of activated NF-κB, we studied NF-κB activity in SHRs. As expected, the present study demonstrated that the renal activity of NF-κB was significantly increased in SHRs. Meanwhile the protein level of IκB-α significantly decreased in the kidneys of the SHR, a finding suggestive of the activation of the canonical NF-κB signal pathway in the setting of hypertensive nephropathy. This canonical signal pathway entails the activation of IκB-α kinase and the subsequent phosphorylation-induced proteolysis of IκB-α inhibitors, phosphorylation of the NF-κB p65 subunit and the nuclear translocation of the active NF-κB complexes, which act as transcription factors [45].

## CONCLUSION

RSV treatment significantly attenuates renal damage in the SHR model of chronic renal disease. The renoprotective effect is associated with inhibition of micro-inflammation cytokines including IL-6, ICAM-1 and MCP-1 via the regulation of the nuclear translocation of NF-κB. Given that RSV has high oral bioavailability and excellent safety profile in human studies and clinical trials [46,47], our studies suggest that RSV may be a potential treatment approach in hypertensive renal damage through its effect on renal fibrosis. The research reported here provides new insight regarding the pathogenesis of CKD and provides a foundation for future clinical trials to treat CKD, a life-threatening disorder with limited therapeutic options.

## Abbreviations

CCR: creatinine clearance rate
CKD: chronic kidney disease
Col I: collagen type I
Fn: fibronectin
ICAM-1: intercellular adhesion molecule-1
IL-6: interleukin-6
MCP-1: monocyte chemoattractant protein 1
β_2_-MG: β_2_-microglobulin
NF-κB: nuclear factor κB
PAI-1: plasminogen activator inhibitor-1
PAS: periodic acid-Schiff
RSV: resveratrol
RT: reverse transcription
SHR: spontaneously hypertensive rat
TGF-β: transforming growth factor-β
TGF-β1: transforming growth factor-β1
TNF-α: tumor necrosis factor-α
WKY: Wistar Kyoto rat
